# 4278 Recovery Time is Exaggerated in Individuals with Degenerative Cervical Myelopathy Following Standing Lateral Waist Pulls

**DOI:** 10.1017/cts.2020.313

**Published:** 2020-07-29

**Authors:** Timothy Boerger, Learon McGinn, Marisa Clare, Marjorie Wang, Brian D Schmit, Allison S Hyngstrom

**Affiliations:** 1 Marquette University; 2 Medical College of Wisconsin

## Abstract

OBJECTIVES/GOALS: The aim of this study was to quantify balance impairments in stance in individuals with degenerative cervical myelopathy (IwDCM) in response to external perturbations. IwDCM have damage to their spinal cord due to degeneration of the cervical vertebral column, but little is known about balance. METHODS/STUDY POPULATION: Recovery time following a perturbation may be an important measure of balance. Changes in recovery time were measured in 7 IwDCM (2m, 58.59±15.00y) and 6 controls without DCM (2m, 56.91±11.04y) as they stood on an instrumented treadmill and received cued (predictable) and uncued (unpredictable) lateral pulls to the waist at 12% (high) and 6% (low) pull magnitudes. Individuals stood with feet together, shoulder width, and wide. Recovery time was defined as the time following pull onset when the absolute value of the center of pressure velocity returned to <1x baseline standard deviation. Repeated measures ANOVA was performed on recovery time. RESULTS/ANTICIPATED RESULTS: We anticipate that feet together standing, unpredictable, higher magnitude perturbations will be most challenging evidenced by longer recovery times. For waist pull recovery time, there was a trend for a Group x Predictability x Magnitude x Stance Width interaction (p = 0.1) which we anticipate being greater with additional participants. There were significant Group x Predictability x Stance Width (p = 0.01) and Group x Magnitude x Predictability (p = 0.01) interactions. IwDCM had exaggerated recovery times in narrow and wide stances with unpredictable pulls. IwDCM recovered more slowly in response to unpredictable higher magnitude pulls. DISCUSSION/SIGNIFICANCE OF IMPACT: Balance responses in IwDCM are most impaired in narrow stances and when perturbations are unpredictable. Rehabilitation should focus on shortening latency of response timing and increasing power utilization during balance response to promote quicker recovery.

